# Supplementary figures and images for: Substantial cold tolerance in all life stages of Culicoides nubeculosus (Diptera: Ceratopogonidae)
Source: J Med Entomol. 2026 Feb 20;63(1):tjag020. doi: 10.1093/jme/tjag020 (PMC13055875; doi:10.1093/jme/tjag020)

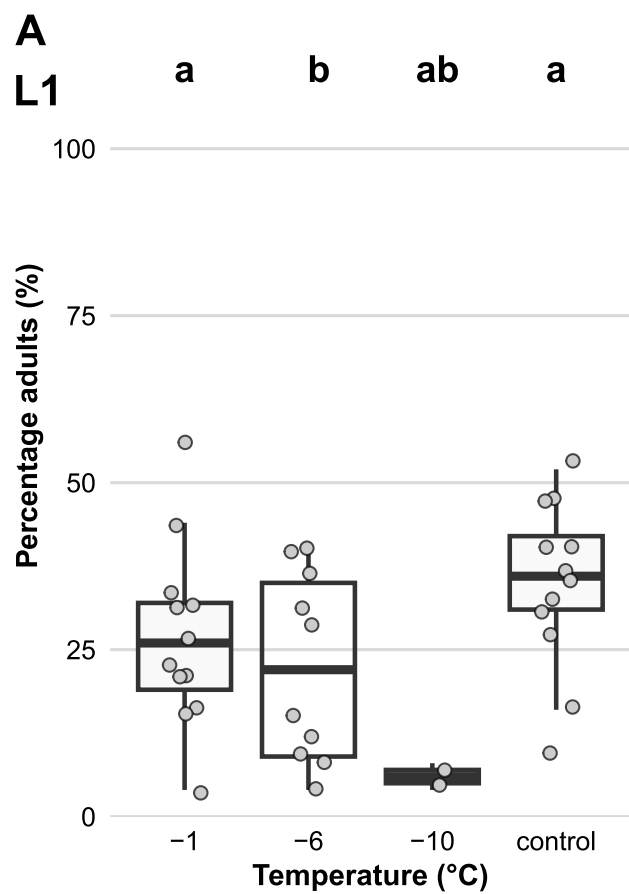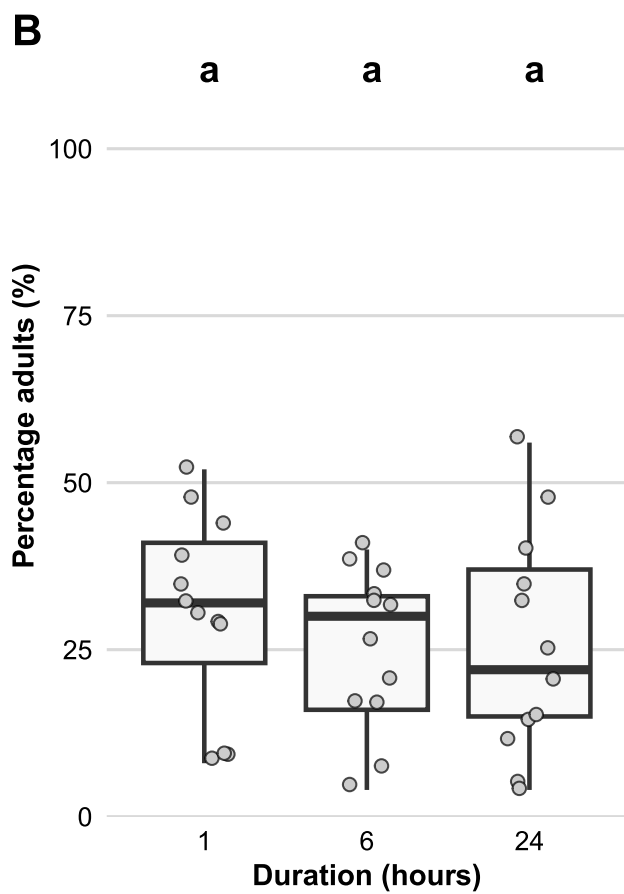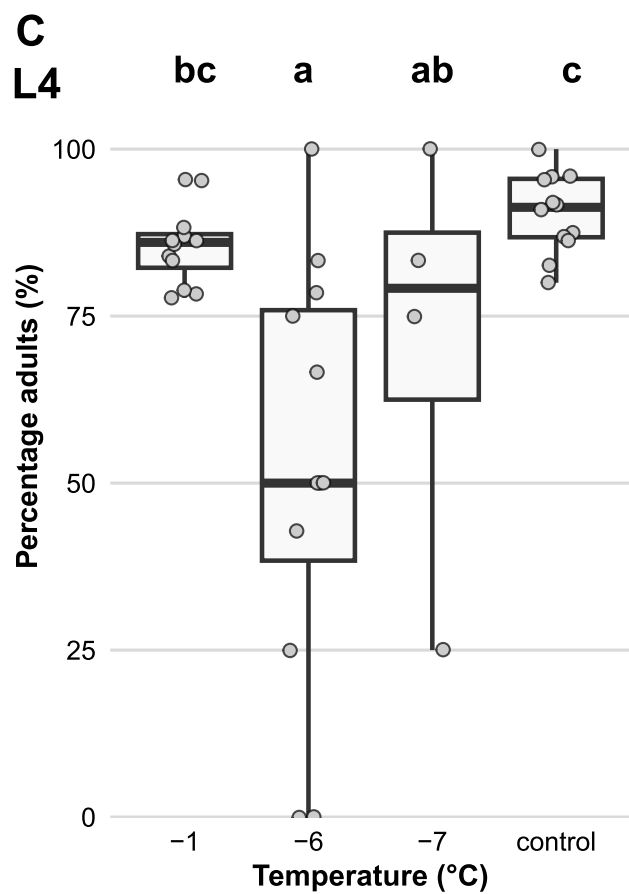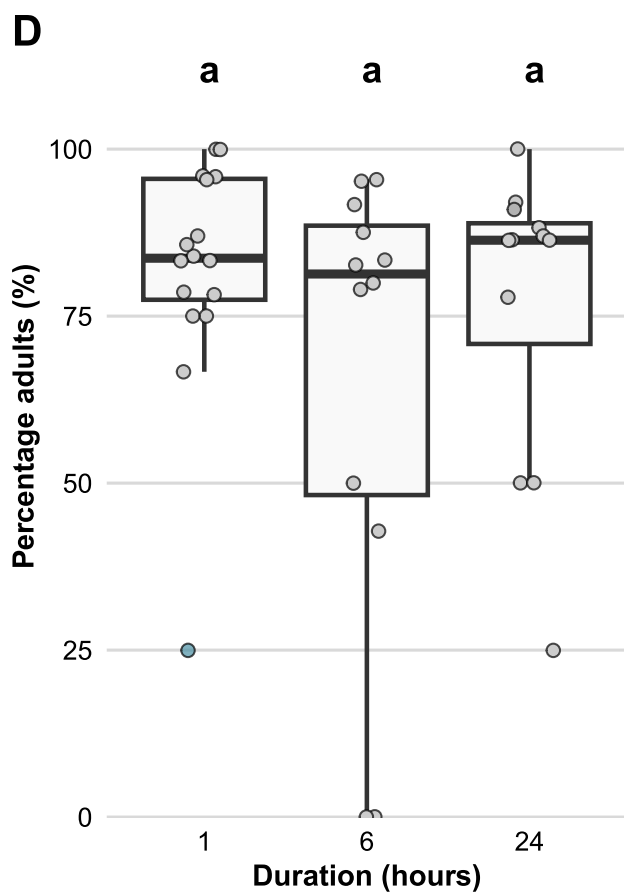

Supplement: tjag020_Supplementary_Data [file tjag020_supplementary_data.zip › Figure_S2.pdf]

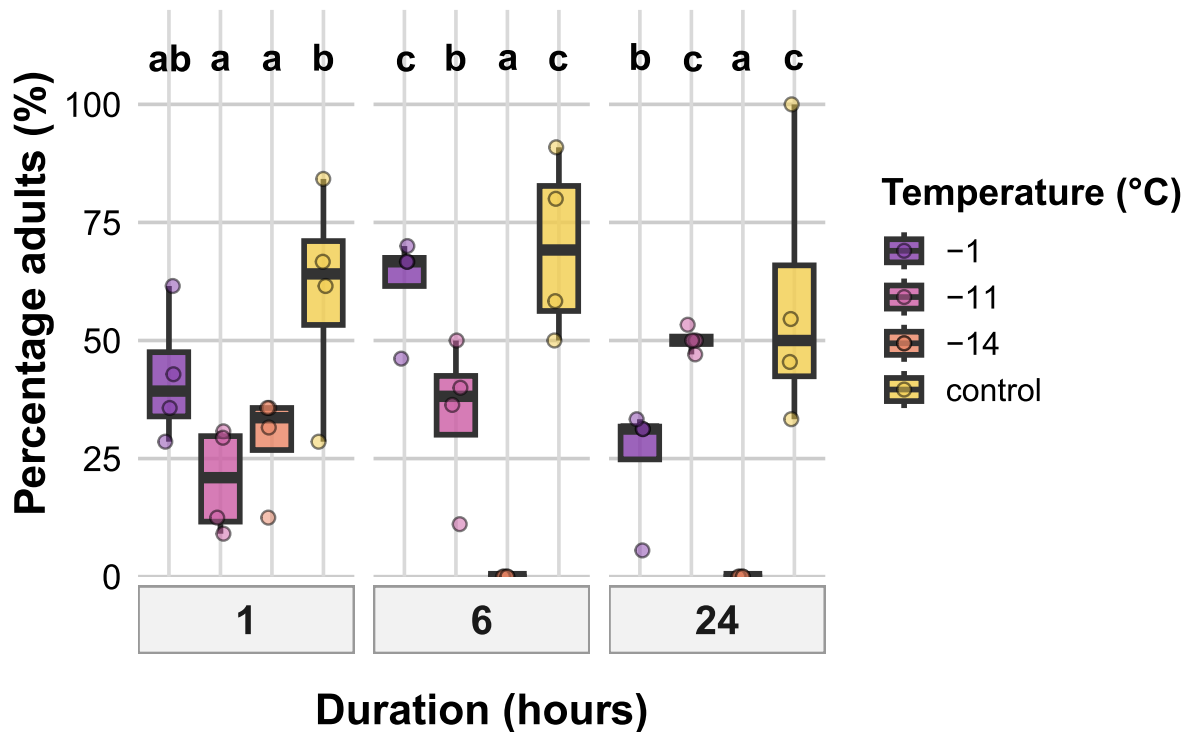

Supplement: tjag020_Supplementary_Data [file tjag020_supplementary_data.zip › Figure_S1.pdf]
